# A guide to null models for animal social network analysis

**DOI:** 10.1111/2041-210X.12772

**Published:** 2017-04-12

**Authors:** Damien R. Farine

**Affiliations:** ^1^ Department of Collective Behaviour Max Planck Institute for Ornithology 78457 Konstanz Germany; ^2^ Chair of Biodiversity and Collective Behaviour Department of Biology University of Konstanz 78457 Konstanz Germany; ^3^ Department of Zoology Edward Grey Institute of Field Ornithology Department of Zoology University of Oxford Oxford OX1 3PS UK

**Keywords:** group living, null model, permutation test, social network analysis, sociality

## Abstract

1. Null models are an important component of the social network analysis toolbox. However, their use in hypothesis testing is still not widespread. Furthermore, several different approaches for constructing null models exist, each with their relative strengths and weaknesses, and often testing different hypotheses.
2. In this study, I highlight why null models are important for robust hypothesis testing in studies of animal social networks. Using simulated data containing a known observation bias, I test how different statistical tests and null models perform if such a bias was unknown.
3. I show that permutations of the raw observational (or ‘pre‐network’) data consistently account for underlying structure in the generated social network, and thus can reduce both type I and type II error rates. However, permutations of pre‐network data remain relatively uncommon in animal social network analysis because they are challenging to implement for certain data types, particularly those from focal follows and GPS tracking.
4. I explain simple routines that can easily be implemented across different types of data, and supply R code that applies each type of null model to the same simulated dataset. The R code can easily be modified to test hypotheses with empirical data. Widespread use of pre‐network data permutation methods will benefit researchers by facilitating robust hypothesis testing.

## Introduction

With increasing interest in the study of animal social behaviour has come a greater reliance on statistical tools tailored to analysing social data. One notable method is social network analysis. An important element in applying social network method to animal data has been the development of specialised hypothesis testing routines (Whitehead 1995, 1999; Bejder, Fletcher & Brager 1998; Lusseau, Whitehead & Gero 2008; Franz & Nunn 2009; Sundaresan, Fischhoff & Dushoff 2009; Farine 2014). One such method is null models, which are used to generate the patterns expected from the data in the absence of the process of interest (Croft *et al*. 2011; Farine & Whitehead 2015). Null models are important because networks are based on non‐independent observations of multiple individuals, and because small differences in how data are collected between individuals can easily generate patterns that appear as social structure. Thus, the aim of constructing a null model is to account for non‐social factors that affect co‐occurrence of individuals (e.g. individual attractions to resources such as bird feeders and watering holes: VanderWaal *et al*. 2013; Adelman *et al*. 2015), so that we can extract the signal of social factors that structure the social network. Social network studies that test hypotheses without such informed null models should be interpreted with caution.

While there are well‐developed and frequently used routines for constructing null models appropriate for social network analysis, these methods were largely designed for observations of randomly and independently sampled groups. However, there is no well‐defined routine for constructing null models using raw data generated from successively following focal individuals and recording their interactions with others, despite this being a common sampling method (Altmann 1974). Furthermore, new animal tracking techniques (Krause *et al*. 2013; Kays *et al*. 2015; Strandburg‐Peshkin *et al*. 2015) are generating new types of data formats that require tailored approaches for implementing null models. In this paper, I explain what null models are, why they are important and how to apply them to social network analysis. I describe recently developed and new null model routines for a range of different data types. I then provide recommendations for how to decide on which null model to use.

### What is a null model?

A null model is any routine that generates datasets against which the observed dataset can be compared (Gotelli & Graves 1996). Routines can include simulations (e.g. creating random graphs) or randomisations of data (permutations). The aim of the null model is to create replicated datasets in which the aspect that is of most interest to us, often who is observed with who, is randomised. At the same time, the model should strive to maintain constant all other aspects of the data that are not directly relevant to the hypothesis, such as the location where individuals were observed. Thus, the aim is to create ‘random’ datasets where only the particular aspect of interest is random, but all else remains equal. The most commonly used with social networks are permutation tests, where the observed data itself are shuffled to create the randomised datasets. The process of shuffling can maintain aspects of the data consistent, while allowing others to change.

### Why null models are needed for animal social network analysis

Social data are inherently non‐independent. For example, for one individual to have three edges connected to it in the network, it requires three other individuals to have at least one edge. Thus, it violates the assumptions of data independence in parametric statistics (Croft *et al*. 2011). Furthermore, testing whether the global structure of a network is non‐random requires something against which to compare the network. A parametric test could only determine whether the network metric differs significantly from zero – a highly unsatisfactory null hypothesis. Finally, null models are a general and powerful approach for testing hypotheses across all levels of social network analysis. They enable us to test specific hypotheses, to explicitly separate out alternative hypotheses, and potentially even to draw comparisons between networks.

### Simple steps for designing null models

#### Design principles

When designing null models, it is useful to consider two questions:

1. What ‘could’ have happened by chance?
2. How would the data look if the process of interest is present or absent?

These questions represent the fundamental foundations of hypothesis testing from both a biological and a data perspective. The aim of the first question is to critically evaluate what the possible outcomes of a set of behaviours in the study species could be. In the context of social networks, this often concerns the distribution of individuals’ social interactions with potential receivers. For example, individuals behaving at random could (i) interact equally with everyone they come into contact with, (ii) come into contact equally with all others they share a home range with or (iii) share a home range with a random set of other individuals. Thus, the distribution of individuals’ interactions, the patterns of contact or the distribution of individuals in space all represent different potential processes that could (or not) have happened by chance. One aim of network analysis is to quantify the minimum contribution that social preference can play in structuring the network, above and beyond factors such as home range overlap or shared use of resources.

The answers to the first question should then guide how we answer the second. Here, we aim to specify how the observed data would be structured if the process(es) identified in part 1 operated at random. For example, individuals with preferences for the same resources could repeatedly be observed together, and thus appear to have social preferences even if they have none. Alternatively, social preferences can drive patterns of space or resource use (Shizuka *et al*. 2014; Firth *et al*. 2015). In the former, the probability of observing two individuals together will be determined by the overlap in their distributions of preference for each resource. In the latter, the probability will be significantly higher than what the preferences alone can explain.

#### Outline of the basic routine

**Figure 1:**
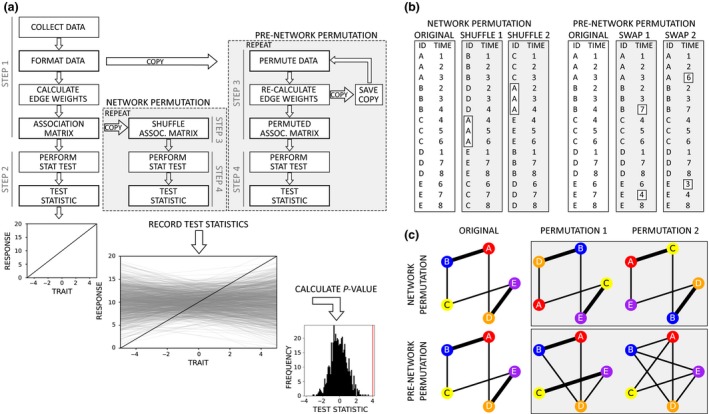
Overview of how to use null models for hypothesis testing. (a) Four main steps involve creating a social network from the observed data, calculating a test statistic (a slope or network‐level metric), randomising the network or the observation data and recording the distribution of possible test statistics. Comparing the observed to the distribution provides the *P*‐value (see Fig. 2). (b) Data perspective of network and pre‐network permutation models. The former randomly swaps all of the data between all individuals (e.g. A gets all of C's data in one permutation, then randomly gets all of B's data in another permutation, see Section ‘Network permutations’). These swaps are typically performed on the adjacency matrix. Pre‐network permutations incrementally swap observations among individuals one at a time (by swapping what time they were observed, such as observations of individuals B and E being swapped so that B now occurs at time 7 while E occurs at time 4, see Section ‘Pre‐network data permutations’). (c) Network perspective of permutation models. Network permutations maintain the same network but change where all individuals are located in the network. Pre‐network data permutations generate increasingly different networks that could reasonably have occurred.

The general process for testing a hypothesis using a permutation‐based null model involves performing four general steps (see Fig. 1):

1. Generate the social network from the observed data
2. Calculate and record the test statistic, using conventional statistics such as linear (mixed effect) models on the data from the observed network
3. Randomise the observed data and generate a ‘random’ social network
4. Calculate and record the test statistic, using the exact same model as in 2, but on the random social network

Step 1 involves making decisions about how to generate networks from the data. There are several useful guides to help with this process (Whitehead 2008; Farine & Whitehead 2015). In general, the decisions made here should have little impact on the choice of the null model used (but may have an impact on the outcomes of the study). For example, the choice of how to quantify the relationship between individuals (e.g. the association index) can influence the distribution of edge weights (Cairns & Schwager 1987; Farine & Strandburg‐Peshkin 2015). However, because the null model will construct the null networks in exactly the same way (including using the same association index), the choice of index should not interact with the choice of null model.

**Figure 2:**
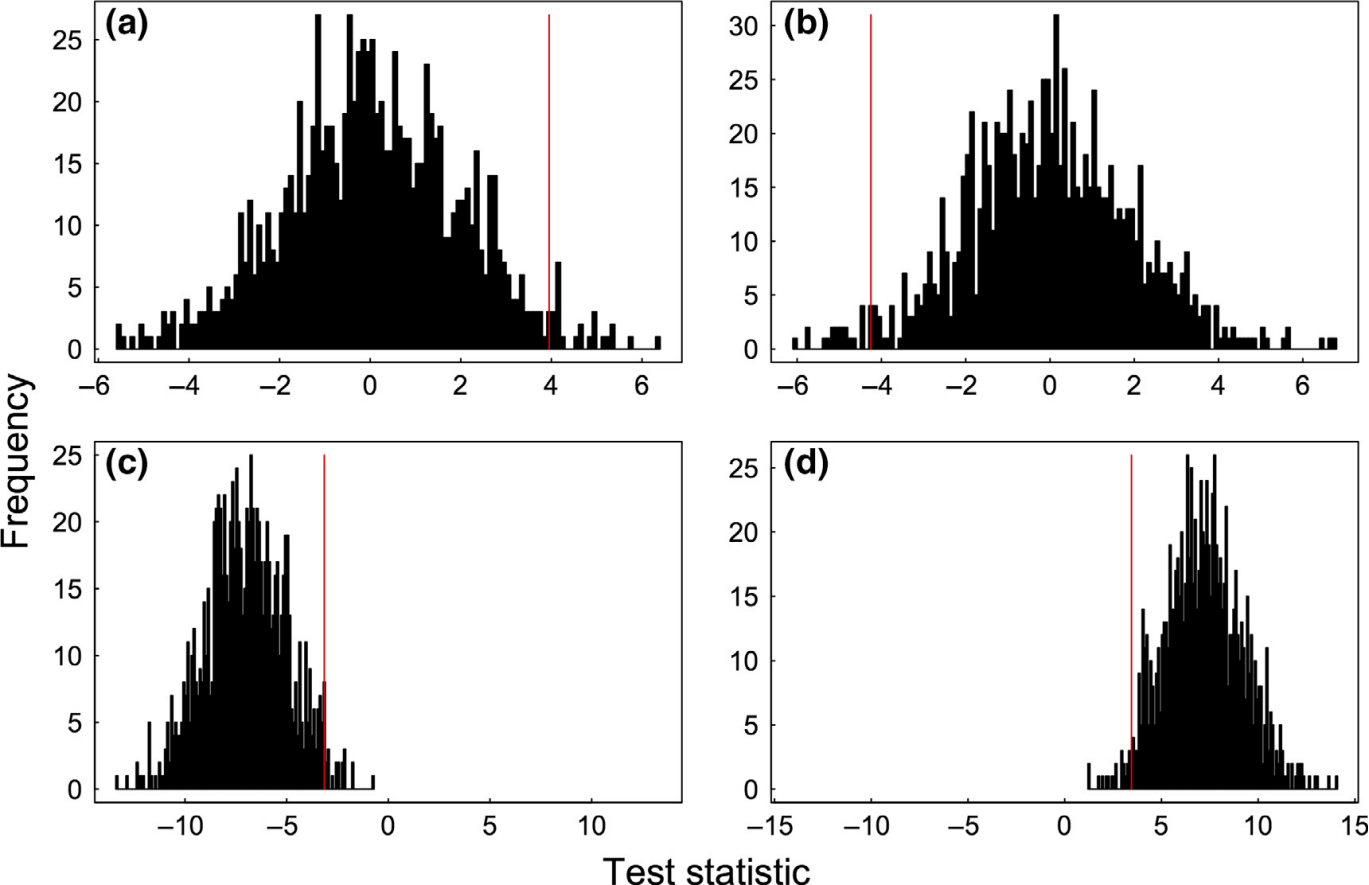
Distributions with all different possible combinations of test results. For most hypothesis being tested, the observed test statistic could be either greater or smaller than random, and two‐tailed values are used. The result is significant at *P* = 0·05 if fewer than 2·5% of the random values are greater than the observed value (a), or if more than 97·5% of the random values are greater than the observed value (b). In many social networks, the distribution of random values will not be centred on 0 (e.g. because individuals are not randomly distributed in space). In these cases, the same logic applies, with significance thresholds (for *P *=* *0·05) when fewer than 2·5% of the random values are greater than the observed (c), or more than 97·5% are greater than observed (d). In two‐tailed tests, the *P* value reported is twice the proportion (e.g. the *P* = 0·05 threshold lies at 0·025), with values close to 1 being subtracted from 1 (e.g. a value of 0·975 or above is significant at *P* = 0·05 as 1−0·975 = 0·025).

In steps 2 and 4, the choice of statistical model will depend on what is appropriate for a particular study. For this paper (and elsewhere, e.g. Farine 2013; Boogert, Farine & Spencer 2014; Farine & Whitehead 2015), I have found that linear or mixed models are useful for extracting test statistics when comparing among nodes in a network. I recommend using the coefficient of the slope as the test statistic when constructing null models rather than the *t* or Z statistics. This is because the coefficient describes the data, whereas the *t* or Z statistics represent the departure of the data from the parametric null hypothesis. To calculate significance of network‐level metrics, the metric itself can be used as the test statistic (e.g. the mean degree or coefficient of variation in edge weights). When using null models, steps 3 and 4 are repeated a large number of times, at least 1000 times, although many more are required if the dataset is large. The best way to determine if enough samples are drawn from the null model is to plot the value of the test statistic vs. the randomisation number to see if the value has stabilised, or to plot the *P* value against randomisation number (see Fig. 2 in Bejder, Fletcher & Brager 1998). The following section deals in detail with how to accomplish step 3. The set of values recorded in step 4 is used to generate the expected distribution of the data if the process was random. We then use this expected distribution to compare the observed data against.

Based on the distribution of test statistic values generated by the null model, we can calculate the significance of the test to determine whether we can reject the null hypothesis. Significance is calculated by determining where the observed test statistic falls relative to the distribution of test statistics calculated using the random networks. This is done by counting the number of times a random test statistic was greater or smaller than the observed test statistic, and dividing this by the number of null datasets that was compared. Figure 2 provides a schematic to guide interpretation.

#### Demonstrating the importance of using null models: a simulation

Farine & Whitehead (2015) demonstrated why null models are important for hypothesis testing in animal social networks using simulated data of social associations among individuals in a population. In their simulation, they introduced an observation bias for females, mimicking a process in which dull females were more easily overlooked during observations than bright males, by removing 20% of the observations of females (i.e. although females were always present, they were only recorded in 80% of the samples). Although there was no statistical difference in the weighted degree between males and females in the original data (before removing 20% of the samples), a parametric test incorrectly rejected the null hypothesis in the observed data, thus claiming that females had a significantly lower degree. However, when using a pre‐network data permutation test (see Section ‘Pre‐network data permutations’), the null hypothesis was not rejected (i.e. avoided a type I error). Because the permutation test was based on the observed data, each version of the permuted data against which the observed data were compared contained the same sampling bias. Thus, females had fewer observations in all permuted version of the data.

**Figure 3:**
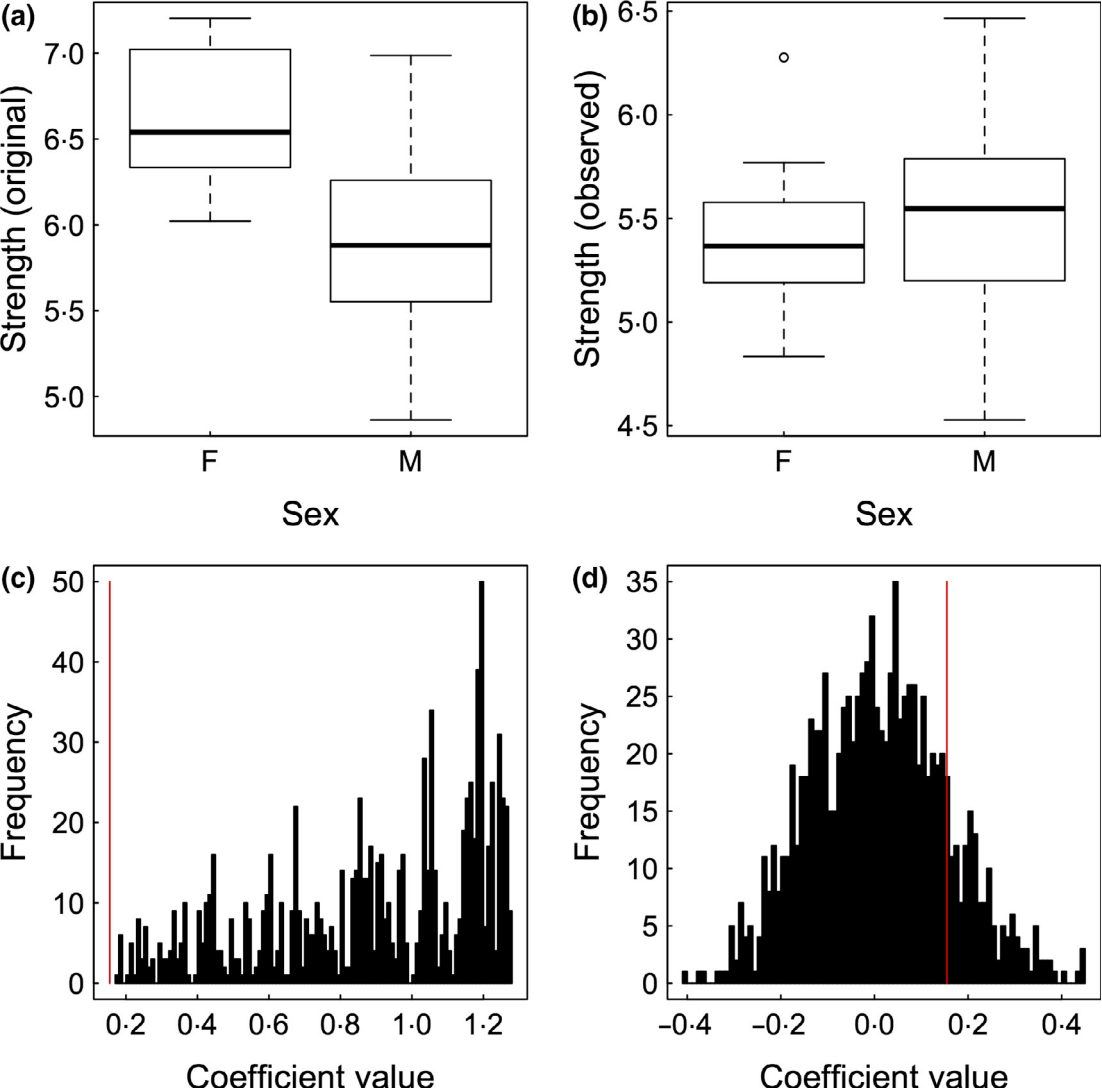
Simulated data demonstrating that pre‐network data permutation tests avoid type II errors in hypothesis testing. (a) A dataset is simulated such that females have a higher weighted degree (strength) than males. (b) After removing a random 20% of data from females, the observed data suggest no difference in weighted degree between the sexes. (c) A pre‐network data permutation test (Section ‘Pre‐network data permutations’) correctly identifies that the observed coefficient's value (red vertical line) is significantly smaller than that expected by chance (the black histogram). (d) By contrast, a node permutation test (Section ‘Network permutations’) does not return the correct result. Both null models used 1000 permutations.

In the simulations by Farine & Whitehead (2015), males and females had similar social phenotypes, but females were observed fewer times. Throughout this paper, I will use a modified simulation where I create social groups in which females truly have stronger bonds (i.e. a higher ‘weighted degree’; Fig. 3a). I then replicate the same observation bias (mimicking a scenario where inconspicuous females are more difficult to observe than ornamented males) and randomly remove 20% of the data from the females (herein the *observed data*). This results in no apparent difference in the weighted degree between males and females (Fig. 3b): applying a regular linear model (Weighted degree ~ Sex) to the observed data suggests that there is no significant difference in weighted degree between the sexes (β = 0·15 ± 0·15, *P* = 0·299) in the observed data. However, this result is subject to a type II error because we know that a difference in sociality exists (a benefit of using simulated data, see Fig. 3a). I note here that in most studies, the number of observations could be fit as a fixed effect in the model, which in this case could fix the coefficient value (although the *P* value could still not be used for inference due to non‐independence issues). However, the aim of this simulation is to demonstrate how null models can directly maintain features of these data rather than having to try controlling for them after generating the networks.

Applying a permutation test that randomises observations from which the social network is inferred (‘pre‐network data permutation test’, see Section ‘Pre‐network data permutations’) correctly identifies that females have a significantly higher weighted degree relative to males when compared to what is expected by chance *given our data* (*P* < 0·01). That is, the coefficient estimates for the effect of sex in the *randomised* data are more positive than the value of the coefficient we calculated for the *observed* data (Fig. 3c). Evaluating the hypothesis using a node permutation test (which randomises the network itself rather than the data on which the network is based, see Section ‘Network permutations’) also fails to account for patterns arising from the sampling bias and return a non‐significant result (*P* = 0·85, Fig. 3d). This example demonstrates that node permutations also suffer from type II errors in these scenarios (as previously noted elsewhere, Croft *et al*. 2011; Farine 2014), and I deal with why this occurs in more detail in Section ‘Network permutations’. To show that this result is not because the null model is always different to the observed data in this simulation, I also provide supplementary R code performing a baseline simulation using the same test on data where there is no difference in weighted degree and no observation bias. In that case, both the pre‐network data permutation and the node permutation models return non‐significant results.

### Constructing permutation tests

There are many different ways that permutations can be implemented. These can generally be classed two categories: network permutations and pre‐network data permutations. The former is performed after the networks are generated, whereas the latter is performed on the data before generating the network (see Fig. 1a). Here, I briefly explain the most common network permutation method (node permutations), and then explain how to perform pre‐network data permutations across a range data types.

#### Network permutations

**Figure 4:**
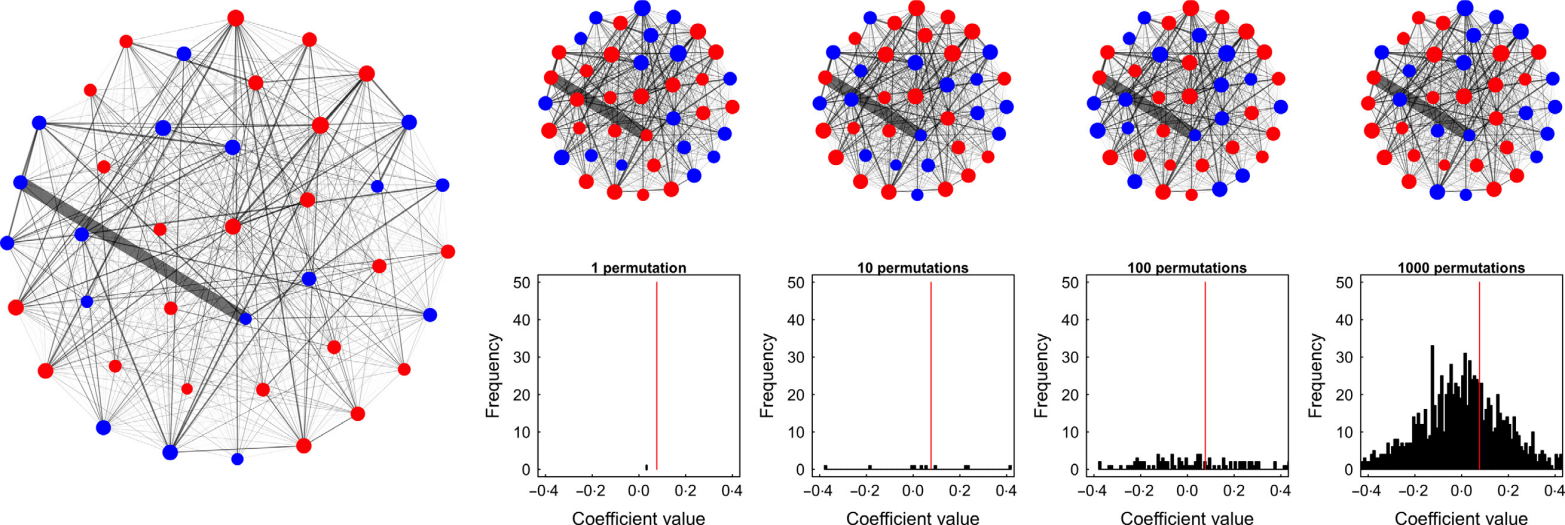
Example of a node permutation. Data are generated as in Fig. 3 to create a social network (left), but where 20% of female observations are removed. In each permutation (*n* = 1000), all the node labels in the original network (red = female, blue = male) are randomly re‐allocated to new nodes, but the network is kept the same. The same model (Weighted degree ~ Sex) is run for each of the permuted networks, which in this case fails to detect a significant effect (see Fig. 3).

The simplest, and perhaps most widely used, permutation test in social network analysis is the node permutation test. In this test, the identity of each node is randomised, thus breaking the link between the network and the traits of interest (the phenotype of each node). This process is most easily achieved by randomising the node labels (i.e. the mapping of the phenotypes to the nodes). For example, take a network containing individuals with sexes [M, M, M, F, F, F]. We construct our network (step 1 in the basic routine), and then (step 2) make a model to test, for example, weighted degree relative to sex in our network (Weighted degree ~ Sex). In a node permutation (step 3), we randomise the phenotypes (now [F, M, F, M, F, M]), and (step 4) fit the same model or calculate the same test statistic as performed on the original data (step 2). We can repeat this randomisation process (steps 3 and 4) many times, creating different versions of the mapping of phenotype to node each time (Fig. 4) but always maintaining the same number of each phenotype and the same network structure.

Node permutations perform a complete swap of all observations (the observations that determine an individual's position in the social network) among individuals (by assigning their identity to a different node). This means that an individual can occupy any position, from the most central (which might generate a positive test statistic) to the most peripheral position (which might generate a negative test statistic). The outcome of this process is that the complete spectrum of potential outcomes is generated by the null model. One way to identify whether a node permutation has been used is that the resulting distribution of test statistics is centred on, or near, zero.

An alternative to node permutations are edge permutations. Here, the edges among individuals are re‐arranged, for example, swapping the edge (or edge values) between A and B to A and C, thus creating new versions of the network each time but always maintaining the same number of edges (and distribution of edge weights if applicable). I do not go into further detail, as their use is relatively uncommon and not applicable to the data I present here, but further discussion can be found in Croft *et al*. (2011).

#### Pre‐network data permutations

**Figure 5:**
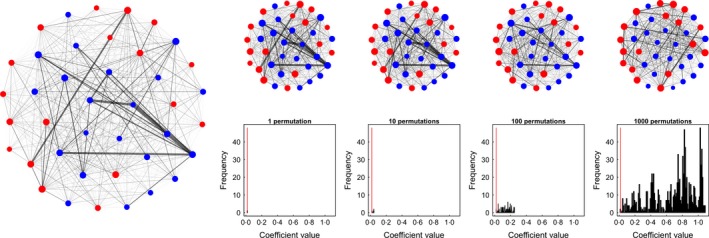
Example of a pre‐network data permutation. Data are generated as in Fig. 3. Observations of two individuals are swapped between groups, thus in this case only slightly changing the edge structure in the social network with each permutation. Because the swaps are performed incrementally, the network after 1 permutation is very similar to the original network, and thus the coefficient does not change much. However, after many swaps, the coefficient of the model on the permuted networks becomes increasingly different from the coefficient estimated from the observed data, with the final result that females in the observed data have a significantly higher degree relative to males than expected. Note that in this case, the ‘random’ coefficient values stabilised between values of 0·8 and 1·0, thus providing evidence that a bias is present in the observation data.

Researchers studying animal social systems realised early on that existing permutation tests were not sufficiently robust for testing many hypotheses of interest. Bejder, Fletcher & Brager (1998) proposed a null model where permutations are performed within the pre‐network data, and thus enabling better control over the model and the hypothesis being tested. The model was originally designed for data where observations are made of animals using the ‘gambit of the group’ approach (Whitehead & Dufault 1999; Franks, Ruxton & James 2010): individuals are recorded as occurring in a social group, and all individuals recorded in the same group are assumed to be associating. In the pre‐network permutation model, single observations of two individuals occurring in different groups are swapped, such that an individual A that occurred in group 1 now occurs in group 2, and individual B that occurred in group 2 now occurs in group 1. Each swap incrementally changes the network a small amount (Fig. 5). The swapping process in this model is often described as finding a ‘checker board’ pattern in the group by individual matrix. A strength of this approach is that it preserves both the number of times individuals were seen, the number and size of groups. Later refinements (Whitehead 1999; Whitehead, Bejder & Ottensmeyer 2005; Sundaresan, Fischhoff & Dushoff 2009) included restricting swaps by time, location, phenotype and a range of other factors. Thus, the swapping algorithm can constrain swaps to occur only between individuals observed at the same place and/or within the same period and/or with the same phenotype. In doing so, the algorithm can control for factors such as home range, observational sampling biases or underlying behavioural differences that might mask the hypothesis being tested.

#### Permuting focal observation data

**Figure 6:**
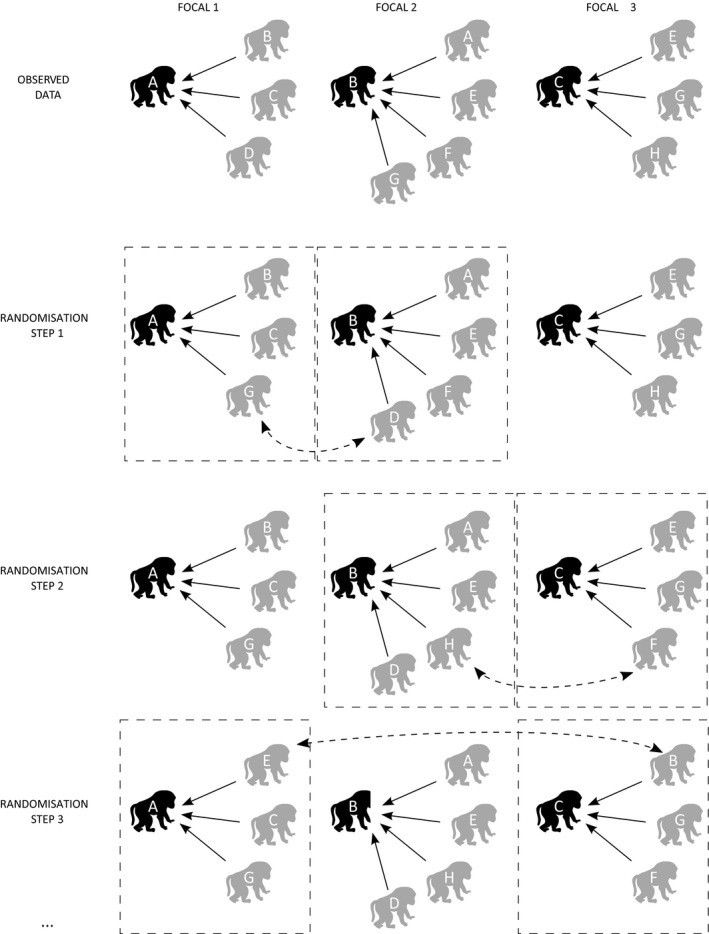
Demonstration of a pre‐network data permutation applied to focal data. For each randomisation, two focal samples are selected at random (dashed boxes), ensuring that these two focal samples are of different focal individuals. One individual from each focal is then chosen and their data are swapped, but only if these individuals do not occur in both sets of observations (including the focal individuals themselves). This process is repeated at least 1000 times, incrementally randomising the data at each step. Note that some, or even all, of the other group members can be the same across the two sets of focal observations between which individuals are swapped, so long as the two focal individuals and the two individuals to be swapped represent four unique individuals.

Focal observations, where a particular individual is followed and its interaction with others are recorded, are a particularly challenging type of data for which to construct null models. The reason is that these data are structured by the focal individual, and randomising data using traditional methods would break this feature of the data. Pre‐network data permutations can be performed by introducing a simple modification: what was a ‘group’ in the pre‐network data permutation method is now the observation of one focal individual with all its associates. The observations of whom two different focal individuals were observed with can now be swapped, taking care to make the extra check that neither of the individuals being swapped are also one of the focal individuals. That is, instead of being swapped between groups, individuals are now swapped between sets of focal observations. This process is illustrated in Fig. 6.

**Figure 7:**
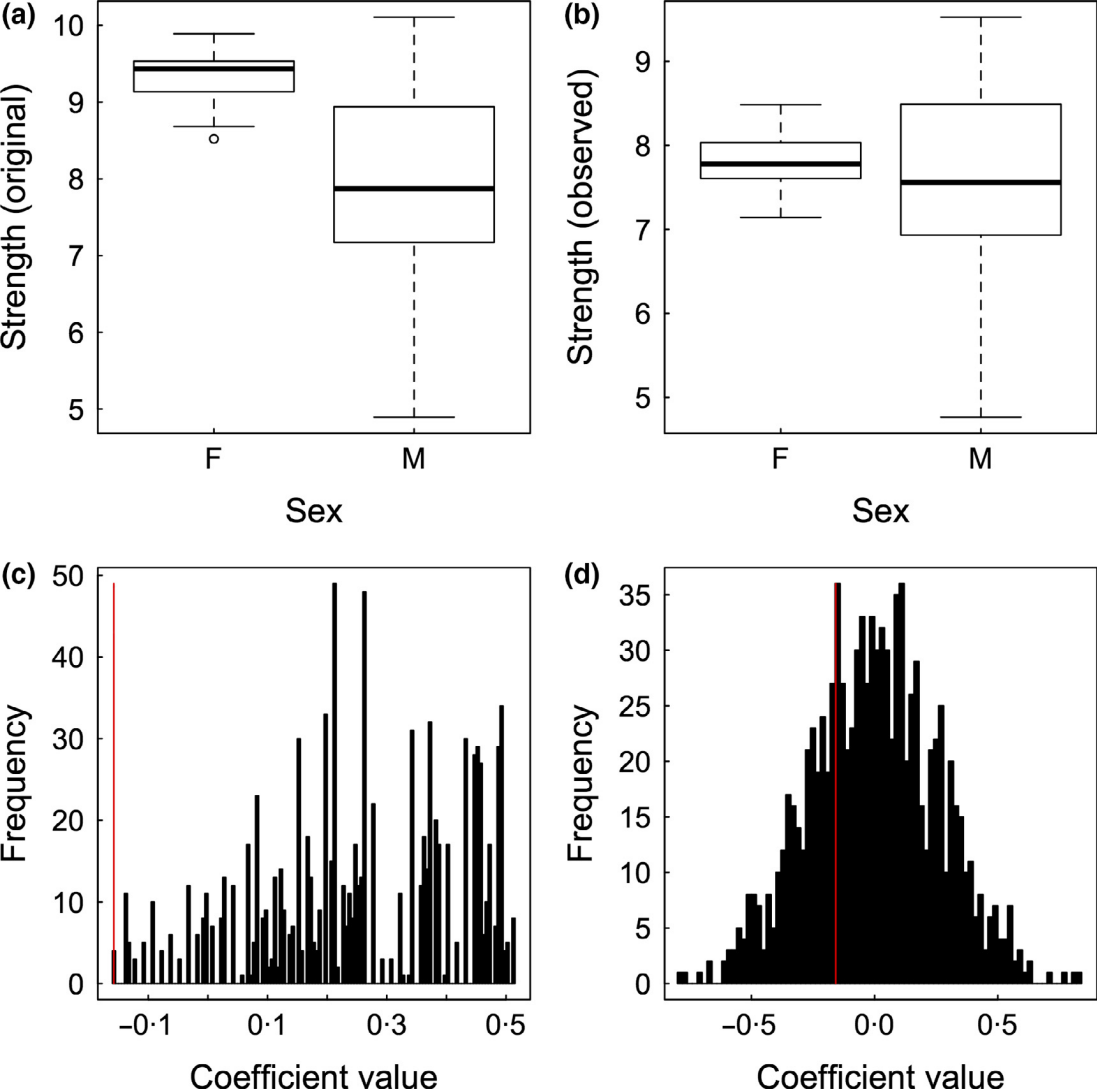
Simulated focal observation data demonstrating the ability for permutation tests to avoid type II errors in hypothesis testing. (a) A dataset is simulated such that females have a higher weighted degree. (b) After removing 20% of data from females, the observed data suggest no difference in degree between the sexes. (c) A focal pre‐network data permutation test correctly identifies that the observed coefficient (red vertical line) is less than expected by chance (the black histogram). (d) By contrast, a node permutation test, which is commonly used on data collected using focal observations, applied to exactly the same data does not return the correct result. Both null models used 1000 permutations.

**Figure 8:**
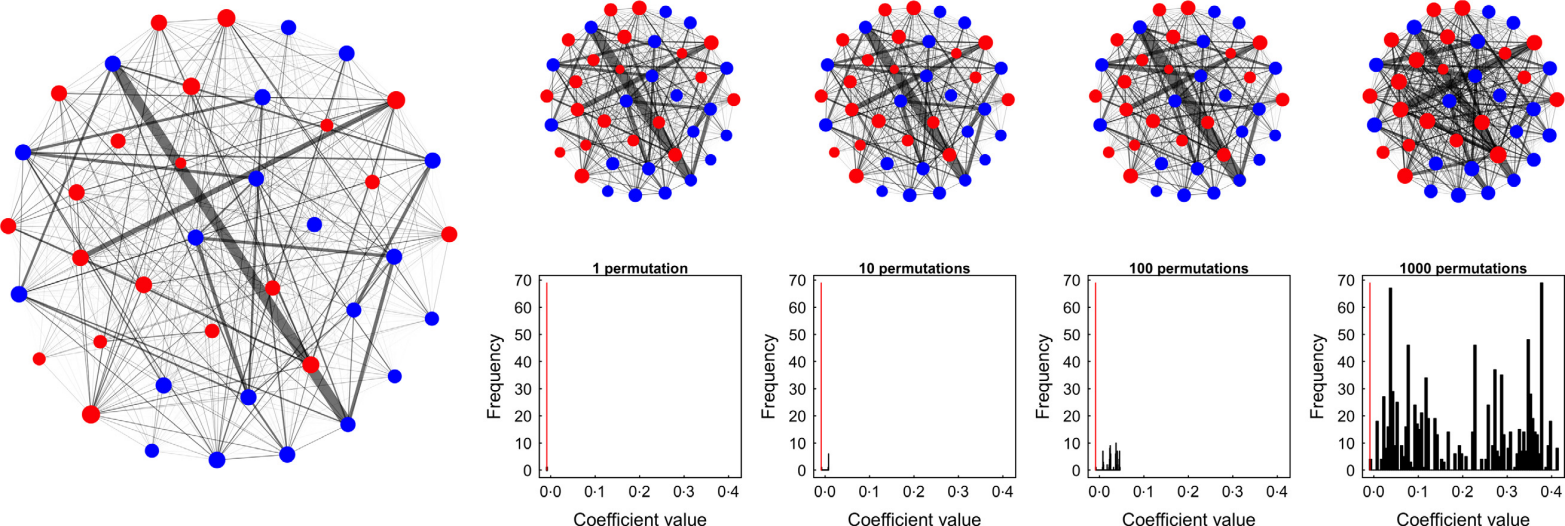
Example of a focal pre‐network data permutation. Data are generated as in Fig. 7 to create a social network (left). In each permutation (1000 performed), observations of two individuals are swapped between sets of focal observations (per Fig. 6), thus only slightly changing the edge structure in the social network.

As with the previously described pre‐network data permutation method (see Section ‘Pre‐network data permutations’), swapping observations in this way maintains the frequency at which individuals occur in the data, and maintains the number of interactions or associations present in each focal observation. To control for temporal features of the data, swaps can be restrained to only occur between focal observations occurring on the same day or similar time period. Restrictions can also be placed on which pairs of individuals are swapped. For example, to control for male–female patterns of affiliations, it is possible to only swap observations between sets of observations where the two focal individuals are both male (or both female). Maintaining such features in the data will allow for precise hypotheses to be tested. In Fig. 7, I demonstrate that pre‐network permutations are useful for focal data. As with pre‐network data permutations, the structure of the edges in the network is only changed slightly after each permutation (Fig. 8).

#### Permuting autocorrelated data streams (e.g. GPS data)

Some data types do not lend themselves to performing pre‐network data stream permutations. For example, researchers tracking animals using GPS generally want to maintain the autocorrelation structure of each individual track, and thus avoid constructing null models that contain tracks with unrealistic individual movements. Several approaches have been suggested to resolve this issue. The general principle has been to segment tracks into discrete chunks (e.g. daily tracks), and perform permutations independently on each of these chunks. However, it is easy to fall into the trap of generating node permutations. Instead, the principles of swapping when individuals were observed (the underlying principle of the Bejder, Fletcher & Brager (1998) method) are a more robust approach.

In a recent study on sleepy lizards (*Tiliqua rugosa*), the social network was constructed from GPS points collected every 10 min (Godfrey *et al*. 2014). To maintain the daily tracks of lizards, a null model was developed such that the identity of each individual was randomised for each day. Thus, the same tracks occurred each day but these were allocated to different individuals, and the identity of the individuals assigned to the tracks differed across days. This appears to be a pre‐network data permutation (because the data are swapped before inferring the network). However, this approach still swapped entire chunks of data among individuals (e.g. the 3 days of observations of individual A are now comprised of data from individuals B, E and H respectively). Thus, it replicated node permutations and resulted in the same limitations.

**Figure 9:**
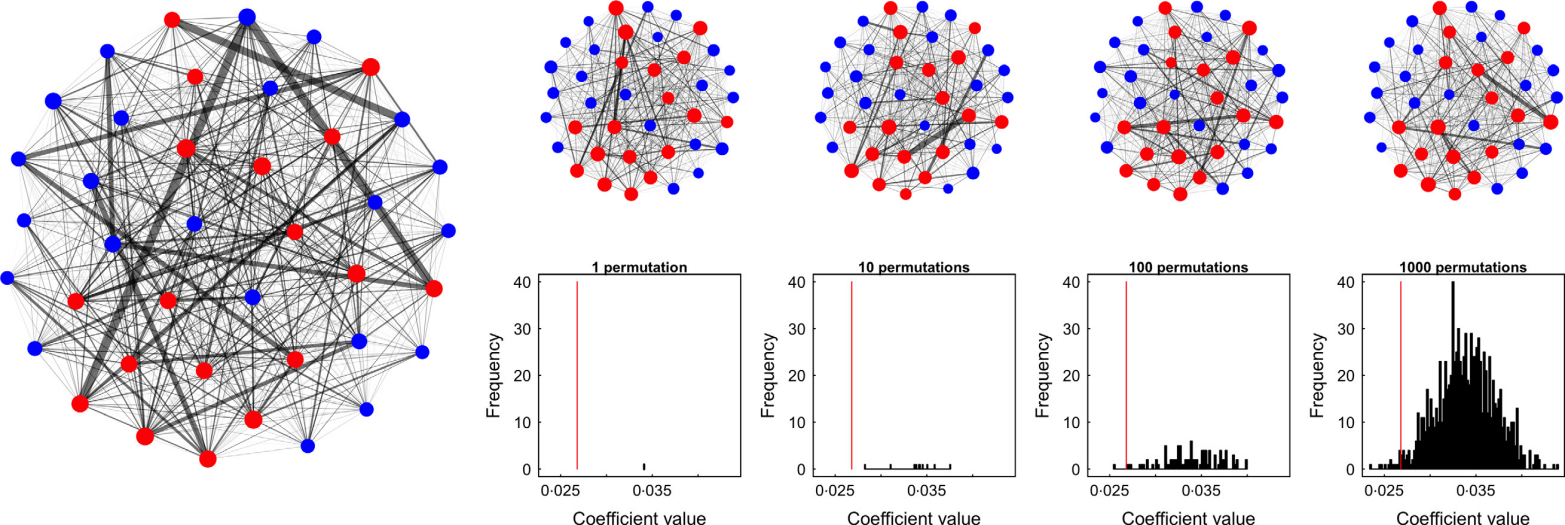
Example of a pre‐network data permutation test applied to GPS data. Individual movements are simulated in an arena where males move at random and females are 5 times more likely to move to a neighbouring cell containing a conspecific than to a neighbouring cell containing no individuals. Once generated, 20% of female observations are removed. In each permutation (1000 performed), the ordering of days for each individual is randomised within individuals. The results of applying this null model are consistent with those from the pre‐network data permutation example (Fig. 5).

In general, we should aim to avoid directly swapping chunks of data among individuals unless this is an explicit aim of the model (see Section ‘Which null model to use?’). In the original pre‐network data permutation model proposed by Bejder, Fletcher & Brager (1998), only the ‘timing’ of focal individuals’ detections were changed (by moving them from one group to another). For studies using GPS tracks to infer networks, Spiegel *et al*. (2016) propose randomising the date of each daily track within individuals instead of swapping data between individuals. The result is that individuals maintain exactly the same spatial data (home range) in the null model as in the observed data, but who they come into contact with (and thus the social network) changes because the order of these data is randomised. In Fig. 9, I confirm that this model has many of the desirable properties of pre‐network data permutations. By contrast, implementing models based on swapping data between individuals do not achieve these same properties (the code for demonstrating this is included in the supplemental R script associated with this figure).

### Alternative methods for hypothesis testing in social networks analysis: current approaches and limitations

In the previous sections, I have shown that null models that maintain as many of the features from the original data as possible are generally better at avoiding erroneous findings. A major implication of this is that many existing approaches for hypothesis testing developed for studies in sociology are likely to have inadequate underlying null models for most hypotheses we are interested in testing. For example, quadratic assignment procedure (QAP) and multiple regression QAP (MRQAP) tests are based on the logic of node permutations, and all simulation‐based approaches, such as exponential random graph models (ERGMs), are based on replicating the observed network (hence making the same assumptions as network permutation methods). Here, I briefly discuss the implications of the findings above on these two approaches.

#### Multiple quadratic assignment procedure

QAP and MRQAP tests are important statistical approaches for hypothesis testing in social network analysis because they evaluate edge‐based hypotheses. Put simply, they perform the equivalent of a regression, where the dependent variable and the independent variables are all networks. In QAP, the significance of the parameter estimate for each independent network is calculated by conducting a node permutation on the dependent network, and comparing the slope of each predictor in the observed model to the slopes generated by the randomisations. MRQAP improves the technique by performing node randomisations on each fixed effect, or its residuals, independently (see Dekker, Krackhardt & Snijders 2007 for further details on why). MRQAP has been used to compare the structure of the social network with the relatedness of each pair of individuals (e.g. Godfrey *et al*. 2014) or to compare patterns of associations with similarity in pathogen presence (e.g. VanderWaal *et al*. 2013).

First, I highlight how the reliance of node permutation approaches in MRQAP can generate type II errors by evaluating the method with the same simulated situation as described throughout this paper. Importantly, MRQAP is used to test hypotheses based on matrices, i.e. Individual by Individual networks, rather than a data table with individual‐level measures, i.e. an Individual by Observation (or N × K) data frame as commonly used in linear models. I therefore simulate data but this time categorise each dyad (pair of individuals) into three sex categories: 1 = both males, 2 = male–female dyads, 3 = both females, creating an N × N matrix of sex categories for each dyad as the predictor variable. I then use MRQAP to test if there is a relationship between the social network and the sex category of each dyad (Association Network ~ Sex Similarity). Because females have a higher degree in the ‘real’ network, we expect a significantly positive relationship between association strength and sex category (edges connected to females should be stronger).

The effect of sex similarity when the model was applied to the simulated data with biased observation was weak (β = −0·003). The standard MRQAP model suggested it was non‐significant (*P* = 0·233). An alternative is to generate a set of ‘null’ networks using a more robust permutation method, and fit these networks to the MRQAP function to generate the null distribution of test statistics. I have implement a function in my r package asnipe (Farine 2013) to enable customised null models to be used in conjunction with a QAP or MRQAP regression. Using a pre‐network data permutation test on the simulated data gave the correct results: *P* = 0·005 (99·5% of the random networks resulted in a β > −0·003). This result is exactly what we expect: females all have a higher degree and males a lower degree, and thus edges connected to females should have higher weights than expected by chance. In the absence of any relationship in the data, the method based on the pre‐network data permutation accurately reports no significant effect (see supplemental baseline code for this method). These results suggest that using pre‐network permutation test with MRQAP warrants further consideration.

#### Exponential random graph models (ERGMs)

ERGMs are used to generate hypotheses about what structural processes underpin the formation of social networks (Snijders *et al*. 2006), and function by randomly adding and removing edges to see how they change the network. The model uses the changes in the network to determine how the different parameters in the model (what we would call ‘fixed effects’ in linear models) predict the changes in the structure of the network. For example, it can test the propensity for two individuals to share the same co‐associates (which is called triadic closure) by evaluating whether removing edges disproportionately disconnects triads (sets of three nodes all connected together). An issue arising with these models is that they work directly with the observed networks, without consideration of underlying uncertainty or data collection methodology. Although ERGMs have been used with animal social networks (Ilany, Booms & Holekamp 2015; Rankin *et al*. 2016), they also do not incorporate a permutation procedure for testing hypotheses.

Using the simulation framework previously described, I generated networks and fit a standard ERGM model (using the r package statnet, Handcock *et al*. 2015) that includes sex as a node effect. This model tests whether edges are disproportionately likely to be connected to females (which we know should be the case because the simulated data are designed that way). The first issue with this approach is that it can only work with binary networks (where edges are 1 or 0). An issue with this approach is it cannot have completely connected networks, despite these being common, such as in primates. I cut the network at the median edge weight, such that the top 50% of edges remain (are 1) and the lower 50% are removed (now 0), but note that thresholding the network is problematic because it is arbitrary and ignores important weak edges (see Farine 2014). The results of the ERGM suggest a significant negative relationship between being female and having connected edges (β ± SE = −0·308 ± 0·104, *P* = 0·003). This is clearly incorrect, as running the same model on the original network (without the removed observations but with the same thresholding applied) shows that females do in fact have a higher degree than expected (β ± SE = 0·410 ± 0·105, *P* = 0·0001). Thus, ERGMs do not presently have the functionality to contrast the observed data to an appropriate null hypothesis.

### Which null model to use?

Throughout this paper I have focused largely on answering questions about the individual drivers of social network structure. For example, a hypothesis might be that individuals with bold personalities have higher binary degree (more associates) in the social network (e.g. Aplin *et al*. 2013). If the null model controls for spatial distribution, then a correlation between boldness and degree suggests that bold individuals have a higher degree than expected within the context of the social environment they experience. By contrast, if the null model does not control for spatial distribution (such as in a standard node permutation), then a correlation suggests that bold individuals have a higher or lower degree than everyone else in the network, but this could be because of where they live as well as because of their behaviour. Thus, controlling for space is important for discrimination between social avoidance and spatial separation (Spiegel *et al*. 2016).

In some cases, it is necessary to maintain the same network structure. In a study on repeatability of social network position in great tits (*Parus major*) detected at feeders using PIT tags, Aplin *et al*. (2015) needed to maintain the variance structure in terms of network positions in their null model, and thus the same distribution of node values in the social network (which is not the case for pre‐network data permutations because the network structure changes). Faced with the issue of having to avoid swapping individuals widely across the study site, the authors swapped data between individuals that were observed at the same location and within the same time period. This null model is still a node permutation (it performs swaps after the network has been inferred), but one controls for variation in density and some other factors that are determined by individuals’ home range by preventing swapping individuals to new locations.

Maintaining the social network constant is also likely to be particularly important for studies that focus on studying processes occurring over the network itself. For example, one could test for correlations between binary degree and acquiring a disease. Using a node permutation will test if individuals that have more associates were more likely to be observed with the disease. In most cases, this is what we are actually interested in finding out. Alternatively, we could be interested in understanding the effects of local behavioural differences on disease risk, in which case a pre‐network data permutation model would test whether the individuals that are relatively more social are also at greater risk of acquiring the disease. A network (node) permutation tests hypotheses at the network level (did the disease spread in the densest part of the network). A pre‐network permutation tests hypotheses at the individual level (did more social individuals acquire the disease). Many studies would benefit from implementing multiple null models (Farine *et al*. 2015), and explicitly testing competing hypotheses.

### Comparing networks

A major challenge for animal social networks is the difficulties with comparing networks (Croft, James & Krause 2008). Many factors, such as the density of the network, the number of nodes and how the network data was collected, can influence network metrics (such as degree). For example, recording associations using the gambit of the group will yield higher density networks than recording interactions, thus creating networks where individuals have a higher average degree as a by‐product. Network comparisons need to be able to disentangle real differences in network structure or metrics from apparent differences due to non‐biological processes. Null models could provide a useful tool for allowing comparisons between networks. For example, permutations can test if the networks differ more or less than expected by chance. In this case, the observed statistic is a measure of difference (e.g. the difference in mean degree). Both networks are then simultaneously randomised (by performing one swap at a time within both networks), and after each step the statistic is recalculated. The *P*‐value is then calculated by comparing the observed difference between the network to the distribution of possible differences (per Fig. 2).

## Conclusions

Null models are an important part of hypothesis testing in animal social networks, and careful consideration is needed when choosing which null model to use. Although pre‐network data permutation models were first described nearly 20 years ago (Bejder, Fletcher & Brager 1998), they are still not as widely used as they should be. I have shown in this paper that null models can be implemented on all data types. I also highlight that we need to continue efforts to further develop existing methods for hypothesis testing to be able to apply them to animal social networks.

## Data accessibility

This study does not include any data. All figures can be generated using the supplemental R scripts.

## Supporting information


**Data S1.** The supplemental information consists of 11 R scripts. Each script refers to a figure that uses simulated data.
